# Microbiota composition data for wild and captive bluestreak cleaner wrasse *Labroides dimidiatus* (Valenciennes, 1839)

**DOI:** 10.1016/j.dib.2020.106120

**Published:** 2020-08-03

**Authors:** Victor Tosin Okomoda, Ashyikin Noor Ahmad Nurul, Abdullah Muhd Danish-Daniel, Abraham Sunday Oladimeji, Ambok Bolong Abol-Munafi, Korede Isaiah Alabi, Asma Ariffin Nur

**Affiliations:** a Department of Fisheries and Aquaculture, College of Forestry and Fisheries, University of Agriculture, P.M.B., 2373 Makurdi, Nigeria; b Institute of Tropical Aquaculture and Fisheries Research (AQUATROP), Universiti Malaysia Terengganu, 21030 Kuala Nerus, Terengganu, Malaysia; c Institute of Marine Biotechnology, Universiti Malaysia Terengganu, 21030 Kuala Nerus, Terengganu, Malaysia; d Faculty of Food Science and Fisheries, Universiti Malaysia Terengganu, 21030 Kuala Nerus, Terengganu, Malaysia.; e Agricultural Department, National Biotechnology Development Agency (NABDA), Abuja, Nigeria.; f Department of Agricultural Extension and Management, Federal College of Forestry, Jos, Plateau, Nigeria

**Keywords:** Bluestreak cleaner wrasse, Ornamental fishes, Microbial analysis, NGS technology

## Abstract

The *Labroides dimidiatus* is known as the “doctor fish” because of its role in removing parasites and infectious pathogens from the body of other fishes. This important role played both in wild and captive conditions could represent a novel form of parasitic transmission process mediated by the cleaning activity of the fish. Yet, there is a paucity of data on the microflora associated with this fish which is important for tracking disease infection and generally monitoring the health status of the fish. This article, therefore, represents the first dataset for the microbiota composition of wild and captive *L. dimidiatus*. Wild fish samples and carriage water were gotten in Terengganu Malaysia around the corals of the Karah Island. The captive sample, however, was obtained from well-known ornamental fish suppliers in Terengganu Malaysia. Thereafter, bacteria present on the skin, in the stomach and the aquarium water were enumerated using culture-independent approaches and Next Generation Sequencing (NGS) technology. Data obtained from the three metagenomic libraries using NGS analysis gave 1,426,740 amplicon sequence reads which are composed of 508 operational taxonomic units (OTUs) for wild samples and 3,238,564 valid reads and 828 OTUs for captive samples. All sequence reads were deposited in the GeneBank (Accession numbers SAMN14260247, SAMN14260248, SAMN14260249, SAMN14260250, SAMN14260251, and SAMN14260252). The dataset presented is associated with the research article “16S rDNA-Based Metagenomic Analysis of Microbial Communities Associated with Wild *Labroides dimidiatus* From Karah Island, Terengganu, Malaysia” [1]. The microbiota data presented in this article can be used to monitor the health and wellbeing of the ornamental fish, especially under captivity, hence preventing possible cross-infection.

**Specifications table**

**Table:** 

Subject	Biological Science
Specific subject area	Aquatic Science
Type of data	Figures and Tables
How data were acquired	DNA extraction and sequencing Microsoft Excel software for computation of bacterial composition
Data format	Raw and Analyzed
Parameters for data collection	Fishes for the study had no physical abnormality or showed signs of stress.
Description of data collection	In this data article, DNA extraction was done from samples of the skin, gut, and carriage water samples collected from the *Labroides dimidiatus* fish gotten from both wild and captive environments. The 16S rRNA gene was obtained from the samples, amplified, sequenced, and deposited in the GeneBank.
Data source location	Institution: Universiti Malaysia Terengganu City/Town/Region: Terengganu Country: Malaysia
Data accessibility	Deposited in the GeneBank (Accession numbers SAMN14260247, SAMN14260248, SAMN14260249, SAMN14260250, SAMN14260251, and SAMN14260252) and With the article
Related research article	A.N.A., Nurul, A., Muhd Danish-Daniel, V.T., Okomoda, A. A. Nur, 2019. 16S rDNA-Based Metagenomic Analysis of Microbial Communities Associated with Wild *Labroides dimidiatus* From Karah Island, Terengganu, Malaysia. Biotechnology Reports. 21: e00303. https://doi.org/10.1016/j.btre.2019.e00303[1]

Value of the Data

• Microbiota associated with *Labroides dimidiatus* was presented for the very first time.
• Data can be used by ornamental fish hobbyists and other scientists working in the area of fish microbiota especially as it relates to ornamental fishes.
• The data is a reference for future studies and useful for comparison with the microbiota of other ornamental fishes obtained from the wild or maintained in captivity.
• Data can assist in the monitoring of the health status of the fish as any substantial variation in the structure as well as the abundance of the bacteria presented in this research can be used as an early sign for disease infection in the species (especially under captivity).
• The analysis of the data as given in the Microsoft excel can serve as a guide for future studies in, processing data for presentation and publication.

## 1 Data description

**Fig. 1:**
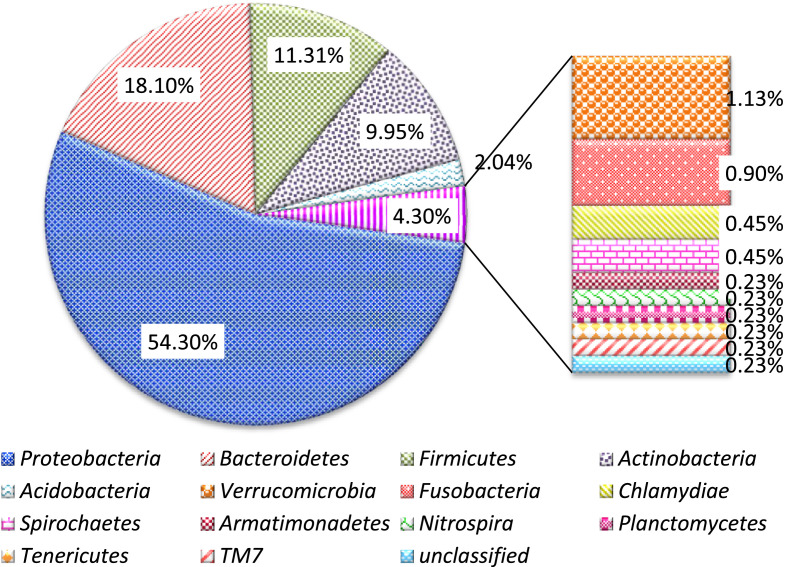
Relative abundance of all bacterial phyla associated with Bluestreak Cleaner Wrasse, *Labroides dimidiatus* from captive and wild environment.

**Fig. 2:**
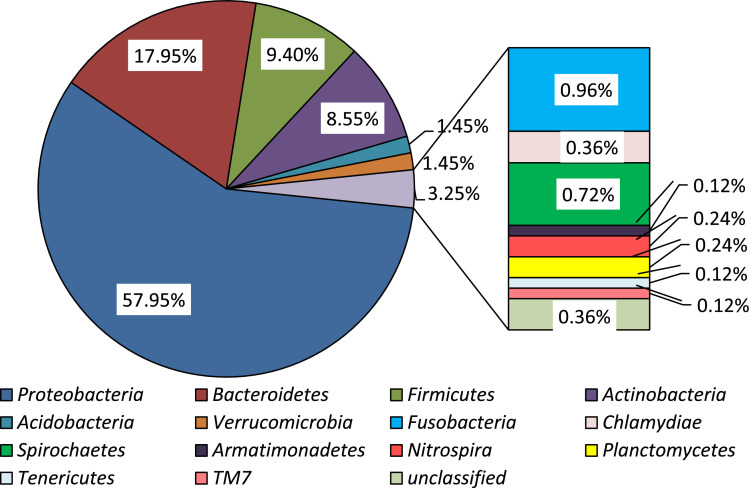
Relative abundance of bacterial phyla associated with Bluestreak Cleaner Wrasse, *Labroides dimidiatus* samples (skin mucus, gut samples, and water samples) obtained from the captive environment (Aquarium).

**Fig. 3:**
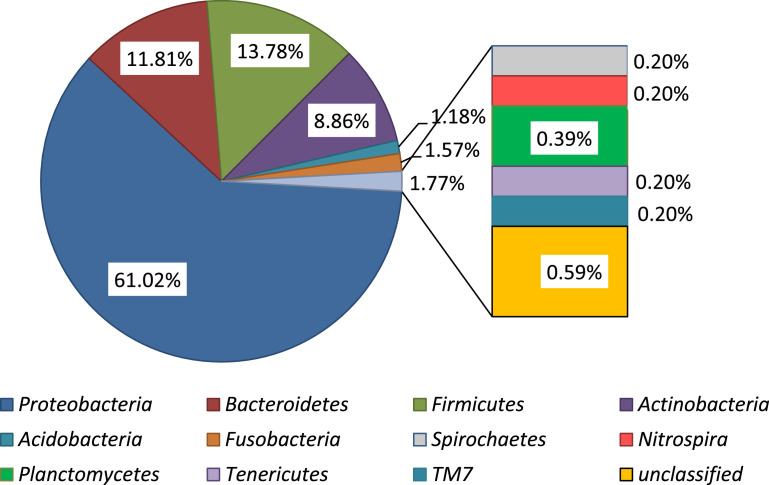
Relative abundance of bacteria phyla in Bluestreak Cleaner Wrasse, *Labroides dimidiatus* samples (skin mucus, gut samples, and water samples) obtained from Karah Island.

**Fig. 4:**
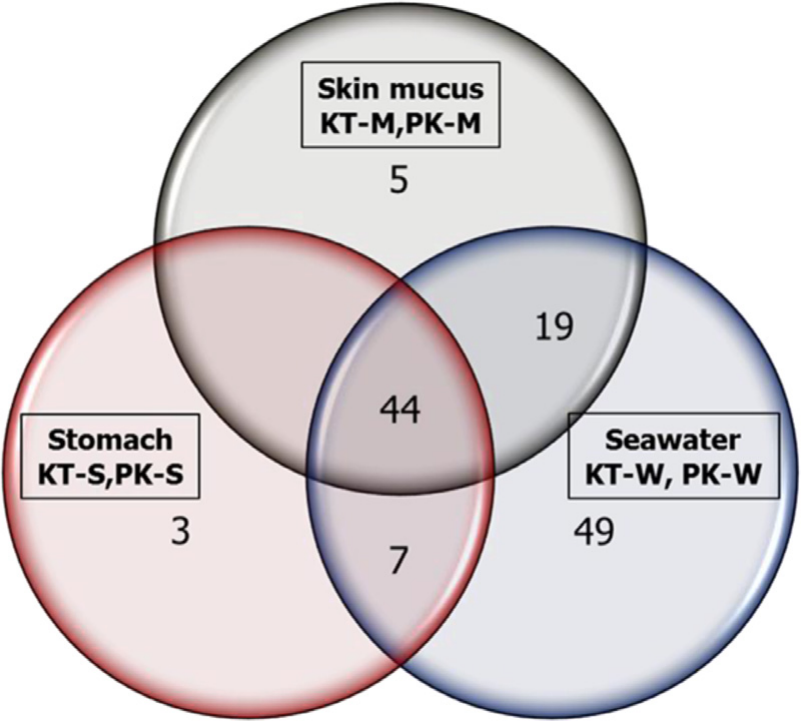
Venn diagram of the common bacteria families of *Labroides dimidiatus* identified in the three different sample types as obtained from the captive and wild environment. KT-M=Skin mucus of fish in captivity; KT-S=Stomach of fish in captivity; KT-W=water sample from aquarium; PK-M=Skin mucus of fish from Karah Island; PK-S=Stomach of fish from Karah Island; PK-W=water sample from Karah Island.

The raw data deposited in the GeneBank represent the sequence reads of the bacterial from the fish skin (SAMN14260247), stomach (SAMN14260248) and carriage water (SAMN14260249) in captivity as well as those from the wild (SAMN14260250, SAMN14260251, and SAMN14260252 respectively). Data presented in the Microsoft excel (Filename: Chart in Microbial of *Labroides dimidiatus*) are the various representations of the compositions in graphs. The percentage of the bacterial phyla associated with *L. dimidiatus* in both environments is presented in Table 1, while the relative abundance of all the bacterial phyla is presented in Figure 1. The bacterial phyla abundance as obtained in the captive and wild environment is as presented in Figs. 2 and 3 respectively. Also, Fig. 4 denotes the vein diagram of numbers of shared and exclusive bacterial families observed in the captive and wild samples of *L. dimidiatus.* Lastly, the standard Illumina forward and reverse primers used for this research are presented in Table 2.

**Table 1:** Bacterial phyla percentages of *Labroides dimidiatus* obtained from wild and captive environment.

Phyla^b^	Sample designation^a^					
	KT-M	KT-S	KT-W	PK-M	PK-S	PK-W
*Proteobacteria*	58.3	62.9	55.4	65.9	60.7	57.7
Bacteroidetes	24.1	10.8	17.9	13.4	7.7	12.8
Firmicutes	7.9	8.2	10.8	10.4	15.4	15.4
Actinobacteria	2.6	12.4	10.0	7.3	12.0	8.4
Fusobacteria	0.9	1.0	1.0	1.8	1.7	1.3
Acidobacteria	1.3	0.5	2.0	0.0	0.9	2.2
Verrucomicrobia	1.8	1.5	1.2	0.0	0.0	0.0
Spirochaetes	0.9	1.0	0.5	0.0	0.9	0.0
Chlamydiae	0.4	1.0	0.0	0.0	0.0	0.0
Unclassified	0.4	0.5	0.2	0.6	0.9	0.4
Nitrospira	0.4	0.0	0.2	0.0	0.0	0.4
Planctomycetes	0.4	0.0	0.2	0.6	0.0	0.4
TM7	0.4	0.0	0.0	0.0	0.0	0.4
Armatimonadetes	0.0	0.0	0.2	0.0	0.0	0.0
Tenericutes	0.0	0.0	0.2	0.0	0.0	0.4

a KT-M=Skin mucus of fish in captivity; KT-S=Stomach of fish in captivity; KT-W=water sample from aquarium; PK-M=Skin mucus of fish from Karah Island; PK-S=Stomach of fish from Karah Island; PK-W=water sample from Karah Island.

b Classification of the Phylum taxonomy and their abundance were based on confidence using the RDP Classifier (%).

**Table 2:** The Primers used for the construction of the Illumina library.

Samples	Primers name	Oligonucleotide sequence (5’–3’)	References
All samples	V3_F	aatcatacggcgaccaccgagatctacactctttccctacacgac gctcttccgatctCCTACGGGAGGCAGCAG	[7]
Skin sample	V3_7R	caagcagaagacggcatacgagat**GATCTG**gtgactggagtt cagacgtgtgctcttcccgatctATTACCGCGGCTGCTGG	[7]
Gut content	V3_5R	caagcagaagacggcatacgagat**CACTGT**gtgactggagtt cagacgtgtgctcttcccgatctATTACCGCGGCTGCTGG	[7]
Water sample	V3_13R	caagcagaagacggcatacgagat**CGTACT**gtgactggagtt cagacgtgtgctcttcccgatctATTACCGCGGCTGCTGG	[7]
All samples	341F	CCTACGGGAGGCAGCAG	[8]
All samples	518R	ATTACCGCGGCTGCTGG	[8]

## 2 Experimental design, materials and methods

*L. dimidiatus* samples with a weight range of 0.5 and 2.8 g were obtained from Terengganu Malaysia. The wild samples were gotten from the coral of the Karah Island while captive samples were obtained from well-known ornamental fish suppliers in Terengganu who had also obtained it from the wild and had maintained them in the aquarium for a month. For the water samples, the collection was done for the ocean and aquarium water in sterilized blue cap bottles (1 L volume), placed on ice [2]. The fish and the carriage water were subsequently taken to the AQUATROP Laboratory for further analysis. In the laboratory, ten healthy fish were killed by pitching after been appropriately tranquilized with tricaine methane sulphonate (MS222) at 150 mg/1 solutions [3]. Skin mucus samples were obtained by dorsolaterally scraping the surface of the dead specimens using an already sterile scalpel [1]. The samples were then processed using the method by Balcázar et al. [4] before storage at −80 °C for further analysis. Also, the same technique used by Balcázar et al. [4] was adopted for gut sample collection, processing, and storage for analysis.

The DNA from the skin and gut samples were extracted using a commercial DNA kit (NucleoSpin® Tissue Kit Machery-Nagel, Germany) without any modification of the manufacturer's protocol. However, water samples were first conditioned according to the method previously used by Wolf et al. [5] before DNA was extracted from them. The amplification of the 16S rRNA gene was achieved using the universal bacteria primer set 63F (5‟-CAGGCCTAACACATGCAAGTC-3‟) and 1389R (5‟-ACGGGCGGTGTGTACAAG-3‟) reported by Hongo et al. [6] following the PCR reaction volume and protocol earlier used by Nurul et al. [1]. In line with the method previously used by Nurul et al., [1], a second PCR was done using 1 μL of the amplicon. Thereafter, the V3 hypervariable region of the 16S rRNA genes was selected according to Bartram et al. [7]. The V3 region amplification of the 16S rRNA gene was then done with the 341F and 518R universal primers reported by Muyzer and de Waal [8]. All the primers used for the construction of the Illumina library are presented in Table 1. Because the V3 specific priming regions were complementary to the standard Illumina primers, they were composed of a 6-bp indexing sequence to allow for multiplexing. The amplification of the primers was then designed with Illumina adapters. PCR amplification condition was according to an earlier report by Nurul et al. [1]. Using gel electrophoresis of 2% agarose, the PCR products were viewed to see if the desired size was gotten and clean-up was done accordingly.

Adapter sequences necessary for binding to the flow cell were denoted by Lowercase letters, while binding sites for the Illumina sequencing primers are the underlined lowercases. Bold uppercase, however, highlighted the indexed sequences while the V3 region primers for the 341F and 518R primers are presented in regular uppercases [7].

The generated “reads” were processed according to the method adopted by Schloss et al. [9] (i.e. trimming and assembling using the software “Mothur”). Overlapping regions within Illumina paired-end reads were aligned to generate “contigs”. The paired-end sequences of a mismatch and those with ambiguous base calls were not used, hence discarded. Thereafter, based on naïve Bayesian classification (RDP classifier) followed by Wang et al. [10], the sequences were assigned taxonomic affiliations. The sequences were then assigned to operational taxonomic units of six samples of the 16S rRNA gene fragments shortly after trimming, screening, and alignment of the same. Thereafter they were connected to the server to download the fastq file. A tab-delimited “oligos” file containing the primer and barcode information was created. Then, the data were analyzed using the Greengenes reference files obtained from the Mothur website. Following the method according to Cole et al. [11], a pairwise similarity cutoff of 97% using the Ribosomal Database Project pyrosequencing pipeline was used to define the operational taxonomic units (OTU) of the bacteria colonies. All the sequence reads generated were deposited in the GeneBank with Accession numbers SAMN14260247, SAMN14260248, SAMN14260249, SAMN14260250, SAMN14260251, and SAMN14260252.

## Ethics statement

The approval for the experimental protocols used for this research was obtained from the Universiti Malaysia Terengganu committee on research. This includes and not limited to methods used for the care and use of animal specimens which were aligned with guidelines of international, national, and institutional standards.

## Declaration of Competing Interest

The authors wish to declare that there are no conflicts of interest whatsoever, be it financial or personal. Hence, none of this was perceived to have influenced the outcome of the research reported herein in this data article.

## Appendix Supplementary materials

Image, application 1

Image, application 2
